# Adipokines in Breast Milk: An Update

**DOI:** 10.4274/jcrpe.1531

**Published:** 2014-12-05

**Authors:** Gönül Çatlı, Nihal Olgaç Dündar, Bumin Nuri Dündar

**Affiliations:** 1 Tepecik Training and Research Hospital, Clinic of Pediatric Endocrinology, İzmir, Turkey; 2 Katip Çelebi University Faculty of Medicine, Department of Pediatric Neurology, İzmir, Turkey; 3 Katip Çelebi University Faculty of Medicine, Department of Pediatric Endocrinology, İzmir, Turkey

**Keywords:** Adipokines, breast milk, infant

## Abstract

Epidemiological surveys indicate that nutrition in infancy is implicated in the long-term tendency to obesity and that a longer duration of breastfeeding is associated with a protective effect against metabolic disorders later in life. However, the precise cause of this association is not well understood. Recent studies on the compounds present in human breast milk have identified various adipokines, including leptin, adiponectin, resistin, obestatin, nesfatin, ghrelin and apelins. Some of these compounds are involved in the regulation of food intake and energy balance. The presence of these adipokines in breast milk suggests that they may be responsible for the regulation of growth in early infancy and that they could influence the energy balance and development of metabolic disorders in childhood and adulthood.

## INTRODUCTION

Human breast milk comprises a variety of nutrients, cytokines, peptides, enzymes, cells, immunoglobulins, proteins and steroids specially suited to meet the needs of newborn infants (1,2). Breast milk has benefits on preventing metabolic disorders and chronic diseases and is referred to as “functional food” due to its roles other than nutrition (1,2). It contains 87-90% water and is the main source of water for newborns (3,4,5). In addition, several peptide/protein hormones have recently been identified in human breast milk, including leptin, adiponectin, resistin, obestatin, nesfatin, irisin, adropin, copeptin, ghrelin, pituitary adenylate cyclase-activating polypeptide, apelins, motilin and cholecystokinin (6,7). These breast milk hormones may transiently regulate the activities of various tissues, including endocrine organs until the endocrine system of the neonate begins to function (6). Some of these peptides are secreted in biologically active forms (3). Leptin, ghrelin, insulin, adiponectin, obestatin, resistin, epidermal growth factor, platelet-derived growth factor and insulin-like growth factor 1 are bioactive substances that play roles in energy intake and regulation of body composition (3). However, functions of some of these peptides in neonatal development are still unknown (4).

Epidemiological studies have shown that breastfeeding has a well-established importance for the infant with a protective role against obesity and metabolic disorders in later life (8,9,10). Previously, breast-fed infants were reported to have less weight gain in the first months of life and that this appeared to be associated with lower obesity risk in childhood and adulthood (11,12). The protective role of breast milk may be attributable not only to its nutritional composition but also to adipokines, which are recently investigated and found to be involved in important physiologic functions (12).

In this review, we present recent data related to adipokines in breast milk and their potential functions.

**Leptin**

Leptin is an adipocyte-derived 167 amino-acid polypeptide hormone discovered in 1994 as the product of the ob gene (Table 1) (6). This hormone is mostly synthesized by the white adipose tissue and the level in the circulation is associated with the amount of body fat mass (2,6). Leptin reduces food intake and increases energy expenditure by acting on the arcuate nucleus through its Ob-receptor (Table 1). Pro-opiomelanocortin neurons producing anorectic α-melanocyte-stimulating hormone (α-MSH) are believed to be the key mediators of leptin action (2). Leptin upregulates the synthesis of anorectic neuropeptides: α-MSH, cocaine-amphetamine-regulated transcript and corticotropin-releasing hormone. Besides, it downregulates the orexigenic neuropeptides: neuropeptide Y, melanin-concentrating hormone, orexins and agouti-related peptide (13). Serum leptin level is a sensor for energy homeostasis. It is well known that leptin influences food intake and energy expenditure in humans (2). Leptin is produced by the human placenta and potentially plays a role in fetal and neonatal growth (14). Umbilical cord leptin levels are reported to be associated with fetal weight, fat mass and body mass index (BMI) (6,15). The early appearance of leptin during fetal life and the recognition of the placenta as another source of its production are findings which suggest that leptin could have an essential role in the control of fetal growth (2).

**Leptin in Breast Milk**

There are a small number of studies which report that leptin is present in breast milk and may be produced by various cell types in the mammary tissue. Smith-Kirwin et al (16) demonstrated that the ob gene is expressed in the mammary gland of lactating women and that mammary epithelial cells produce leptin. In addition, leptin acts as a paracrine factor on the proliferation, differentiation and/or apoptosis of mammary epithelial cells (17,18,19). On the other hand, in an experimental study, Casabiell et al (20) showed that leptin is transported from maternal circulation to breast milk and that it subsequently passes to neonatal blood, suggesting that maternal leptin may exert biological effects on the infant. These two mechanisms may account for the presence of leptin in breast milk (6,17,18,19,20,21).

Leptin receptors have been identified in the gastric epithelial and small intestinal absorptive cells, suggesting that leptin could pass from ingested milk to infant blood and might play a role in the short term regulation of feeding (22). In a study on neonate rats, milk leptin absorption by the stomach was observed to be particularly high during the first half of the lactation period, when stomach production of leptin was still low. During the second half of the lactation period and in parallel with the maturation of the gastric mucosa, the absorption of exogenous leptin was decreased, whereas its endogenous production by the gastric mucosa was increased (23). The absorption of leptin by the immature stomach, especially during the early stages of the lactation period could, at least in rats, emphasizes the importance of the amount of leptin supplied during this period on body weight control in later life (24).

**Factors Affecting the Function of Leptin in Breast Milk**

**Figure 1:**
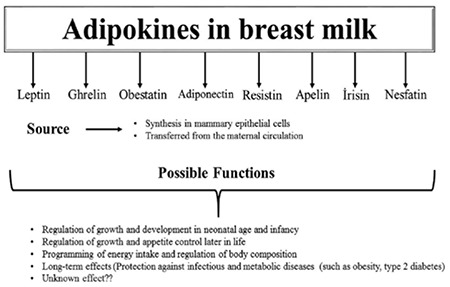
Source and possible functions of breast milk adipokines

Leptin is produced by the human placenta and is potentially involved in fetal and neonatal growth. As many functions of the placenta are changed over by the mammary gland after birth (16), the existence of leptin in breast milk might have a significant role in growth, food intake and regulation of satiety in infants during the early lactation period (Figure 1) (18). It has been proposed that many substances in breast milk are essential for development of the neonatal small intestine or play important roles in immune mechanisms. Leptin also has specific effects on T-lymphocyte responses, such as proliferation of naive and memory T-cells and on increasing IL-2 production and IFN- secretion (25). Previously, leptin receptors were identified on gastric mucous cells which gave rise to the idea that leptin might have a paracrine effect (26). These observations suggest that leptin in breast milk may also have various functions related to gastrointestinal or immune systems (21).

Milk production undergoes three phases. Colostrum is the initial postpartum milk produced in the first 5 days of life, transitional milk is secreted 6-15 days postpartum and mature milk starts to be produced 15 days after delivery (27). Comparative studies of the concentrations of hormones and growth factors have shown that the highest concentrations occur in colostrum (28). Similarly, breast milk leptin level is higher in colostrum than in transitional milk (17) and is decreased during the first 180 days, showing a significant inverse relation with the ongoing days of lactation (29). In addition, it was found at higher levels in whole milk (2-66 fold) than in skimmed samples of breast milk, probably because leptin is associated with milk fat globules (19).

Several studies demonstrated that circulating leptin levels of mothers are positively correlated with maternal BMI and maternal cortisol and thyroxine levels; however, contradictory results have also been published (19,21,29,30). In addition, a positive correlation has also been shown between breast milk leptin and infant plasma leptin levels (30). Leptin is also present in preterm human breast milk at similar levels to that in term breast milk, although lower levels have been detected after preterm compared with term delivery (31). Previously, several studies have investigated serum leptin concentrations in breast-fed and formula-fed infants in the first months of life and found a higher serum leptin concentration in breast-fed infants and a positive correlation between serum leptin concentration and maternal BMI (18,32,33). Savino et al (33) emphasized that a higher maternal BMI might increase leptin levels in breast milk and that maternal adiposity could be involved in infant energy balance. Miralles et al (34) showed breast milk leptin levels to be significantly lower in maternal plasma in non-obese mothers during the whole lactation period and a positive correlation was also demonstrated between leptin levels in breast milk and maternal plasma leptin levels at each of the measured time points (1, 3, 6 and 9 months). In addition, it was shown that maternal BMI correlated positively with plasma leptin concentration and with breast milk leptin level at each of the measured time points (1, 3, 6 and 9 months). Furthermore, breast milk leptin concentration at the first month of lactation was negatively correlated with infant BMI at 18 and 24 months of age (34). In this study, the authors suggested that human breast milk might regulate weight gain during infancy and that the leptin content in breast milk provided moderate protection for infants from excess weight gain and thus lowered the risk of childhood obesity (Figure 1) (34). Previously, Dundar et al (35) have longitudinally investigated the relationship between breast milk leptin levels and weight gain of small for gestational age (SGA), large for gestational age (LGA) and appropriate for gestational age (AGA) infants. They found different breast milk leptin levels in mothers of SGA, LGA and AGA infants. Maternal milk leptin levels were significantly lower in SGA infants and dramatically increased at the end of the first month of life with a more rapid growth compared to AGA and LGA infants. The authors have suggested that the production of leptin in breast tissue might be regulated physiologically according to the needs and state of the infant (35).

**Adiponectin**

Adiponectin (also known as 30-kDa adipocyte complement-related protein; Acrp30) is an adipocyte-derived hormone, which regulates lipid and glucose metabolism, improves insulin sensitivity and fatty acid oxidation and inhibits hepatic glucose production (anti-diabetic and anti-atherogenic effects) (2,36,37,38). Besides, adiponectin has strong anti-inflammatory properties affecting the vascular endothelium such as inhibiting local pro-inflammatory signals, preventing pre-atherogenic plaque formation and inhibiting arterial wall thickening. In addition to its above-mentioned peripheral actions, adiponectin has a central activity in the regulation of energy homeostasis, stimulating food intake and in reducing energy expenditure (Table 1) (38,39).

Adiponectin synthesis is regulated by the peroxisomal proliferator-activated receptor-γ (PPAR-γ), a nuclear receptor whose expression in liver and muscle seems to be important in the mediation of obesity-related insulin resistance (40). Adiponectin has two different receptors: Adipo-R1 is abundantly expressed in skeletal muscle, while Adipo-R2 is predominantly expressed in the liver (Table 1). Suppression of both Adipo-R1 and Adipo-R2 expression inhibits adiponectin binding and decreases PPAR-γ ligand activity, fatty acid oxidation and glucose uptake (37).

Recent studies have demonstrated that circulating adiponectin occurs in three distinct isomeric forms: trimeric low molecular weight, hexameric medium molecular weight and high molecular-weight (HMW) adiponectin, each of which have different biological activities, including improvement of insulin sensitivity and metabolic control and suppression of inflammation. Among these isomeric forms, HMW adiponectin is the most active form in exerting metabolic functions (41). Serum adiponectin level is negatively correlated with the degree of adiposity. In humans, it was shown that low serum adiponectin levels are associated with obesity, type 2 diabetes, dyslipidemia and cardiovascular disease (37,38,42,43,44,45).

**Source and Factors Affecting Adiponectin in Breast Milk**

In 2006 Martin et al (46) and Bronsky et al (47) were the first authors to report the presence of immunoreactive adiponectin in human breast milk (Table 1). Thereafter, several studies demonstrated that breast milk adiponectin levels are negatively associated with the duration of lactation, but positively associated with maternal adiposity (46,48). However, Ozarda et al (49) have reported conflicting results indicating that the adiponectin level in breast milk increases over time during lactation and is affected by the maternal hormonal and inflammatory status. Adiponectin exists in breast milk, with an average concentration of approximately 19 ng/mL (range, 4.2 to 87.9 ng/mL), which is more than 40 times greater than the reported concentrations for ghrelin and leptin (46,47). Adiponectin was reported to be more abundant in cord blood (30.6 mg/L) than in either breast milk (10.9 mg/L) or maternal serum (8.6 mg/L) (43). HMW adiponectin is the most abundant form present in human breast milk (41). Savino et al (50) showed that the adiponectin level in human breast milk positively correlates with the serum levels of adiponectin of both mothers and infants. Besides, Dundar et al (51) have observed no association of adiponectin level in colostrum with birth weight of the infants or maternal BMI.

**Function of Adiponectin in Breast Milk**

The presence of adiponectin in breast milk, the expression of Adipo-R1 in the small intestine of neonatal mice (52) and the expressions of Adipo-R1 and Adipo-R2 in human colon epithelium (Table 1) (53) suggested that not only adipocyte-derived adiponectin but also adiponectin in breast milk and cord blood might play a key role in infant growth and development (6). Weyermann et al (54) found that high levels of adiponectin in breast milk were associated with overweight at the age of 2 years in infants who were breast-fed for at least 6 months. Brunner et al (55) investigated the relationship of adiponectin in breast milk with infant weight gain and body composition up to the age of 2 years. They showed that breast milk adiponectin tended to inversely correlate with early infant anthropometry up to 4 months, but beyond that age and up to 2 years, it was positively associated with weight gain and the sum of skinfolds. Recent evidence support that adiponectin could play a significant role in the regulation of infant growth during lactation in the early weeks of life (41).

**Resistin**

Resistin (also called FIZZ3) is an adipocyte-derived cytokine, which was discovered in 2001 (Table 1) (43,56). It is a regulator for glucose homeostasis and antagonizes the action of insulin in peripheral tissues (in vitro and in vivo), inhibits adipocyte differentiation and may function as a feedback regulator of adipogenesis (2,56,57). In humans, resistin levels were reported to be higher in obese subjects (58). Resistin has been involved in the development of insulin resistance in mice. However, the effects of resistin on insulin sensitivity and adipogenesis in humans, particularly in the perinatal period, are not clear (59). Besides, the role of resistin in obesity-associated insulin resistance is yet controversial because additional evidence suggests that obesity and insulin resistance are related to decreased resistin expression (2).

**Source and Factors Affecting Resistin Levels in Breast Milk**

Ilcol et al (60) first identified resistin in human breast milk in 2008 (Table 1). They found that during lactation, resistin level in breast milk decreases with time and its level ranges from 1710±68 pg/mL at 1-3 postpartum days to 670±18 pg/mL at 91-180 postpartum days. Moreover, resistin levels in both milk and serum of breastfeeding mothers correlated positively with maternal serum estradiol, progesterone, prolactin, thyroxine, triiodothyronine, cortisol, leptin and C-reactive protein concentrations. They also found that resistin levels were higher in the serum of breast-fed infants as compared to levels in either breast milk or their breastfeeding mothers (60). In 2012, Savino et al (61) showed that serum resistin level of breast-fed infants positively correlated with breast milk resistin levels, while no correlations were present between serum and breast milk resistin levels and the anthropometric parameters of the infants or their mothers.

**Function of Resistin in Breast Milk**

Resistin is suggested to link obesity with diabetes. Its physiologic role in humans is still under debate and very little is known regarding the potential function of resistin in children, especially in newborn infants (56,62). Resistin has been shown to be associated with insulin resistance in obese mice. However, in humans, resistin has not been associated with insulin resistance or obesity and the determination of resistin as a marker of insulin resistance in children is not recommended (63,64). In the perinatal period, it seems that resistin is not directly involved in the regulation of insulin sensitivity or adipogenesis (59). Savino et al (65) suggested that resistin could have a role in controlling fetal growth and similar to the other breast milk hormones, could be involved in appetite regulation and in the metabolic development of infants. Moreover, it was advanced that it plays a role in controlling body weight through effective regulation of adipogenesis by negative feedback. The role of resistin in fetal and infantile growth remains to be elucidated (Figure 1) (66).

Serum resistin levels were found to be higher in term than in preterm infants and these levels were reported to correlate positively with gestational age and birth weight, suggesting a possible role for this hormone in regulating energy metabolism and adiposity in utero. Higher circulating resistin levels in term neonates could be advantageous to the infant by facilitating hepatic glucose production and preventing hypoglycemia after birth (62). In a previous study, cord blood levels of resistin and postnatal alterations in serum resistin levels in term AGA neonates have been investigated, showing high resistin levels at birth (8.63±2.94 ng/mL), similar to those on the 4th day of life (7.87±4.02 ng/mL). These findings suggest that this hormone may play a role in maintenance of metabolic neonatal homeostasis (67,68).

**Ghrelin**

Ghrelin is a 28-amino-acid peptide discovered in 1999. It is synthesized predominantly by the stomach (X/A-like endocrine cells), but also by many other tissues such as pituitary, hypothalamus, bowel, lung, heart, pancreas, kidney, placenta and testis (69). Ghrelin is one of the most important orexigenic peptides currently known (Table 1) (70). Two forms of ghrelin have been described: acylated and deacylated ghrelin. The acylated form (known as active ghrelin) is thought to be crucial for binding to the growth hormone (GH) secretagogue receptor 1a (GHS-R 1a) (71). Ghrelin shows strong GH-releasing activity, which is mediated by activation of the GHS-R 1a in humans (71). GHS-R 1a is expressed predominantly in the pituitary and hypothalamus. In addition to the GH-releasing activity, ghrelin has extended physiological actions in the regulation of food intake, gastric motility, gastric acid secretion, modulation of insulin secretion (reduce insulin secretion), adipogenesis (long-term regulation of body weight), cardiovascular function, cell proliferation, bone metabolism, reproduction, glucose and lipid metabolism and immune regulation (anti-inflammatory effects) (65,70,72). Ghrelin stimulates appetite and food intake in rats and humans (73), by acting primarily on the arcuate nucleus of the hypothalamus (74). Ghrelin secretion is increased by fasting and in response to weight loss, while it decreases under positive energy-balance conditions, such as food intake and obesity. Administration of ghrelin stimulates food intake and reduces fat utilization and energy expenditure thus resulting in weight gain and adiposity in rodents (75). Considering that ghrelin is involved both in short-term regulation of food intake and in long-term regulation of body-weight, the presence of this hormone in breast milk could be one of the factors through which breast-feeding may influence infant feeding behavior and body composition later in life (65).

**Source and Factors Affecting Ghrelin Levels in Breast Milk**

Ghrelin is present in both term and preterm human breast milk (76). The active form is 24-fold higher than the inactive form in breast milk (77). It passes from maternal plasma to the milk (51,78), but it is also produced and secreted by the breast tissue (Figure 1) (51). Free ghrelin levels in breast milk are higher than those found in maternal serum and cord blood (51). Its levels are higher in whole milk than in skimmed milk (79) and increase during lactation (80). Kierson et al (79) measured median ghrelin level in whole breast milk as 2125 pg/mL (range, 260-6000 pg/mL), while Savino et al (81) measured median (inter-quartile range) ghrelin level in breast milk as 526.4 (439.86) pg/mL. Colostrum, transitional and mature milk all contain ghrelin. The ghrelin level in these fluids increases gradually and concomitantly with increasing plasma ghrelin levels after delivery (78). Aydin et al (78) showed lower levels of ghrelin in colostrum (70.3±18 pg/mL, range 70-135 pg/mL) than in both transitional milk (83.8±18 pg/mL) and mature milk (97.3±13pg/mL). A further study by Aydin et al (77), in which breast milk ghrelin levels were measured using high-performance liquid chromatography (HPLC), showed that these levels ranged from 128 to 571 pg/mL. Active ghrelin levels increase during lactation and are significantly related to serum ghrelin levels in breast-fed infants. Active and total ghrelin levels in breast milk were lowest (450±25 and 880±80 pg/mL, respectively) at 0-3 days, whereas they increased progressively to 801±43 during 180 days of lactation period and to 3250±380 pg/mL at 91-180 days postpartum (80).

**Function of Ghrelin in Breast Milk**

It is suggested that ghrelin is involved in postnatal growth. Cesur et al (82) reported that active ghrelin level in breast milk at the 4th month of lactation significantly and positively correlated with weight gain of the infants during the study period. Ghrelin in breast milk also seems to be related to the growth of infants during early postnatal life (82). Furthermore, Dundar et al (51) showed that ghrelin in colostrum appears to be related to the anthropometry of infants even at birth. Savino et al (83) observed significantly higher serum ghrelin levels in formula-fed compared to breast-fed infants. They suggested that formula fed infants received a higher amount of ghrelin, thus it was possible that they had a greater feeding stimulus than breast-fed infants and a consequent increase in weight and growth rate (Figure 1) (83,84).

**Obestatin**

Obestatin is a gut hormone and is known to be involved in modulation of eating behavior (70). It is a 23-amino-acid anorexigenic peptide derived from the ghrelin peptide precursor pre-pro-ghrelin and produced by the human stomach, small intestine (70,85) and salivary glands (86). Obestatin is also involved in energy balance regulation and antagonizes the actions of ghrelin (76). The association of obestatin with energy balance regulation has been validated only in animal studies. A novel role for obestatin in adipocyte function and glucose metabolism has been reported in an animal model, suggesting a potential therapeutic standpoint for insulin resistance and metabolic disorders (87). It was proposed that obestatin reduced food intake, regulated body weight gain and gastric emptying and suppressed intestinal motility (Table 1) (43,78,81). In addition, obestatin was reported to inhibit thirst and anxiety, to improve memory, regulate sleep, induce cell proliferation and to increase exocrine pancreatic secretion (88). However, the source, secretion and regulation of obestatin in early infancy and its effects on growth remain to be elucidated (70).

**Source and Factors Affecting Obestatin Levels in Breast Milk**

The source of obestatin in breast milk is not clear, but it may drain through the mammary glands into the milk (Figure 1). Aydin and colleagues first identified obestatin in breast milk in 2008 (76). They have evaluated obestatin levels in serum and milk from 31 lactating women on the second day (colostrum) and the 25th day (mature milk) after delivery and found higher hormone levels in breast milk than in blood. Obestatin levels in colostrum and mature milk were more than twice the corresponding blood levels (76). In another study, serum obestatin levels were measured as 844.87 (805.14) pg/mL in infants, 759.105 (855.55) pg/mL in lactating mothers and as 846.6 (472.07) pg/mL in breast milk (81).

**Function of Obestatin in Breast Milk**

Clinical and experimental confirmation of the metabolic effects of obestatin is still lacking. Aydin et al (76) hypothesized that higher obestatin concentrations in colostrum might suppress a newborn’s appetite in order to prepare the gastrointestinal tract (bowel maturation) to receive milk. Finally, it was suggested that obestatin in breast milk could be involved in growth, appetite and food intake regulation during early life (82).

**Nesfatin**

Oh-I et al (89) first identified Nesfatin-1/Nucleobindin 2 (NUCB2) as an appetite-regulating neuropeptide in 2006 (Table 1). It is associated with melanocortin signalling pathway in the hypothalamus. Nesfatin-1 is expressed in several tissues, including neurons (hypothalamic paraventricular nucleus, supraoptic nucleus, arcuate nucleus, lateral hypothalamic area, nucleus tractus solitarius and spinal cord) and peripheral tissues (pancreas, liver, subcutaneous and visceral fat tissues, brown adipose tissue, skeletal muscles) (89,90,91,92). It is derived from the precursor protein, NUCB2, which is cleaved post-translationally by prohormone convertases into an N-terminal fragment, nesfatin-1 (82 amino acids) and two C-terminal peptides, nesfatin-2 and nesfatin-3. Among the three forms of nesfatin, nesfatin-1 has the most important physiological roles such as regulating appetite and production of body fat (89,90,91). It was shown that intracerebroventricular injection of nesfatin-1 decreased food intake, while injection of an antibody neutralizing nesfatin-1 stimulated appetite (89).

**Source and Factors Affecting Nesfatin Levels in Breast Milk**

Nesfatin-1 was first identified in breast milk of healthy lactating women and women with gestational diabetes (93). In this study, no significant correlation between BMI and serum or milk nesfatin-1 was demonstrated. However, the authors showed a positive correlation between the levels of nesfatin-1 in serum, colostrum and mature breast milk. This study demonstrated that nesfatin-1 was present in human breast milk, but the source of nesfatin-1 in milk is still unknown. The colostrum nesfatin-1 level was measured as 1.1±0.3 ng/dL in healthy lactating subjects.

**Function of Nesfatin in Breast Milk**

The role of nesfatin-1 in breast milk in infant metabolism is still an unexplored subject.

**Apelin**

Apelin, an endogenous ligand for the G-protein-coupled APJ receptor, was originally discovered in bovine stomach extracts by Tatemoto et al (Table 1) (94). The apelin gene encodes a pre-proprotein of 77 amino acids with a signal peptide in the N-terminal region. After cleavage of the signal peptide, several active apelin forms occur such as apelin-36, apelin-17, apelin-13 and apelin-12 (95). Apelin-12 is one of the most potent C-terminal fragments of the polypeptide that possesses a high affinity to APJ receptor and bioactivity in vivo (95,96). Each of the fragments is present in various tissues, including blood and is expressed in various organs, including the gastrointestinal tract, heart, lung, liver, adipose tissue, brain, adrenal glands, kidney and endothelium and is also present in human plasma (97,98). Both mouse and human adipocytes express and secrete apelin (99). They have important physiological roles and are involved in the regulation of cardiovascular and fluid homeostasis, food intake, cell proliferation and angiogenesis (100,101). It has been shown that apelin regulates glucose-stimulated insulin secretion and is involved in glucose homeostasis (Table 1) (102,103,104). Apelin injection can improve glucose tolerance and glucose utilization in insulin-resistant mice (103,105). It has also been reported that insulin sensitivity was diminished in apelin-knockout mice, but could be restored by the injection of apelin (106). Ma et al (102) found that apelin expression and circulating apelin concentrations are increased in obese, insulin-resistant animals and humans and suggested plasma apelin to be a novel biomarker for predicting type 2 diabetes in men.

**Source and Factors Affecting Apelin Levels in Breast Milk**

The mammary gland of the rat contains high levels of pre-proapelin mRNA and protein (76), which are markedly increased during pregnancy and lactation (107). Wang et al (108) demonstrated that the human mammary gland also contains high levels of pre-proapelin mRNA and apelin protein. Aydin et al (77) first identified apelin-13 and apelin-36 in human breast milk in lactating women with gestational diabetes mellitus and in healthy lactating women. During the colostral period, serum apelin concentration was significantly lower in subjects with gestational diabetes mellitus than healthy lactating subjects. In this study, significant correlations between apelin levels in colostrum and mature milk and between those in serum and mature milk were shown. The study could not demonstrate the origin of apelin-13 and apelin-36 in breast milk. In healthy lactating subjects, colostrum apelin-36 and apelin-13 levels were 6.2±1.9 ng/dL and 5.4±1.8 ng/mL, respectively (77).

**Function of Apelin in Breast Milk**

The function of apelin in breast milk is still unknown in infants.

**Conclusion**

Epidemiological surveys have shown that breastfeeding can have a positive impact on the health of the offspring, an impact which is not limited to infancy but extends into childhood and adulthood. Presence of adipokines in human milk, which are substances involved in the regulation of food intake and energy balance, suggests that breast milk is a source of compounds which are of critical importance in the metabolic development of the infant. Considering the association of certain adipokines with adiposity, it is possible to hypothesize that these peptides can influence the programming of energy intake and regulation of body composition leading to a protective effect against metabolic diseases such as obesity, insulin resistance and type 2 diabetes mellitus in later life. Large longitudinal studies on breast-fed infants are required to better understand the long-term effects of different adipokines in breast milk on metabolism and to confirm this hypothesis. Sources, features and possible functions of adipokines in the breast milk are summarized in Table 1 and Figure 1.

## Figures and Tables

**Table 1:**
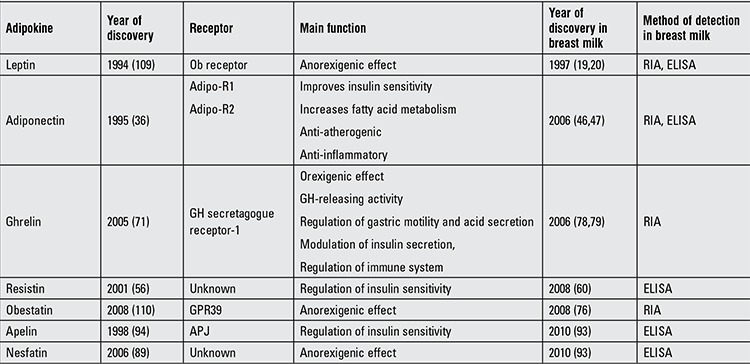
Breast milk adipokines
